# Estimation of the Burden of Iron Deficiency Anemia in France from Iron Intake: Methodological Approach

**DOI:** 10.3390/nu11092045

**Published:** 2019-09-01

**Authors:** Juliana De Oliveira Mota, Patrick Tounian, Sandrine Guillou, Fabrice Pierre, Jeanne-Marie Membré

**Affiliations:** 1 Secalim, INRA, Oniris, Université Bretagne Loire, 44307 Nantes, France; 2 Service de nutrition et gastroentérologie pédiatriques, hôpital Trousseau, Sorbonne Université, AP–HP, 75012 Paris, France; 3 INRA, ToxAlim (Research Centre in Food Toxicology), Université de Toulouse, INRA, ENVT, INP-Purpan, UPS, 31027 Toulouse, France

**Keywords:** iron deficiency, anemia, risk assessment, probabilistic model, second-order Monte Carlo simulation, DALY

## Abstract

Dietary iron deficiency (ID) is the first nutritional deficiency in the world, in terms of disability adjusted life years (DALY). This nutritional deficiency may lead to anemia, especially among children, adolescents, and adult women. The aim of this study was to build an original probabilistic model to quantitatively assess the ID, the iron deficiency anemia (IDA) and the subsequent health burden in France expressed in DALY, per age class and gender. The model considered the distribution of absorbed iron intake, the iron requirement distribution established by the European Food Safety Authority and the iron status in France. Uncertainty due to lack of data and variability due to biological diversity were taken into account and separated using a second-order Monte Carlo procedure. A total of 1290 (95% CI = 1230–1350) IDA cases corresponding to 16 (95% CI = 11–20) DALY were estimated per 100,000 individuals per year. The major contributors to IDA burden were menstruating females aged from 25 to 44 years old. Then, a consumption scenario was built with ground beef as intake, an increase in red meat consumption to 100 g/d would not eliminate entirely the IDA burden. The quantitative methodology applied here for France could be reused for other populations.

## 1. Introduction

The World Health Organization (WHO) estimated that around two billion people were anemic in the world, which makes this disease one of the most common nutrition disorders [1]. Anemia was defined as a hemoglobin (Hb) concentration below the thresholds given by WHO, United Nations Children’s Fund (UNICEF), United Nations University (UNU) [2]. For children aged from 6 to 59 months of age and pregnant women, anemia is diagnosed when Hb is under 11 g/dL, under 11.5 g/dL for children aged 5–11, and under 12 g/dL for children aged 12–14 and non-pregnant females. Finally, for males aged 15 years and above, anemia is diagnosed when the level of Hb is under 13 g/dL. The main cause of anemia identified was iron deficiency (ID), which is defined by WHO as “a state in which there is insufficient iron to maintain the normal physiological function of tissues such as the blood, brain, and muscles” [1]. ID is characterized by no mobilized iron stores and signs of a compromised supply of iron [3]. ID was estimated to represent over than 60% of the anemia causes [4,5], especially among the younger population [4,6]. This nutritional deficiency—commonly defined as serum ferritin level on blood ≤30 µg/L [7,8]—has several consequences for human health, such as a delay in children’s mental and physical development, a decrease in work productivity, intelligence or cognitive capacity [1,9]. In some countries, ID and iron deficiency anemia (IDA) may also contribute to child, maternal, and perinatal mortality [1,9]. Among the overall population, the most concerned by this nutritional disease are children, adolescents, and women (pregnant and non-pregnant) in reproductive age [4,5,10].

To reduce ID, the consumption of bioavailable iron-rich food—such as red meat—would be a logically feasible solution, since this food is the richest source of high absorbable iron (heme-iron) [11]. A study revealed that an increase in red meat consumption twice a week compared to seldom intake would decrease 24% of child ID in Israel [12]. Another study estimated an increase of 0.6% in serum ferritin in blood levels per 1g of red meat consumed [13].

Since 1990, the Global Burden of Diseases (GBD) study, supported by the World Health Organization, has estimated the disability adjusted life years (DALY) associated with health effects of major diseases, injury, and risk factors. This metric takes into account mortality and morbidity and is expressed as the sum of the number of years of life lost (YLL) from premature death and the number of years lived with disability (YLD) [14]. In addition to quantifying the impact of disease, the burden of diseases helps in risk-mitigating strategy: Several consumption patterns can be evaluated and compared by scenario analysis. In particular, in the study of Kassebaum on behalf of GBD 2013 Anemia Collaborators evaluated the global burden of anemia [4]. This work estimated that, in 2013, anemia was responsible for 61.5 (41.0–88.7) millions of YLD in the world, from which 300,654 (200,463–434,060) YLD in France. However, this burden was estimated from hemoglobin levels, which makes scenarios of iron consumption impossible.

Regarding the separation of uncertainty—due to the lack of data and knowledge and variability associated with heterogeneity within populations—[15,16] of the inputs, it is important to identify which of them are driving the output of the risk model and to identify the needed data to increase the precision and the confidence of the estimated output [17].

The objective of this study was to estimate the burden of disease in France due to ID, taking iron consumption into account. Based upon dietary surveys, iron recommendations and blood iron status of the French population, a probabilistic model was set up for both genders and for specific age classes to quantify the risk of ID and the consequent burden of disease due to IDA. Then, to reduce the burden, consumption scenario was built with ground beef intake as red meat is the richest source of high absorbable iron in developed countries.

## 2. Methods

### 2.1. Model Framework for Iron Deficiency Anemia Assessment

The model was developed for males and females aged from three years old for which the age classes were 3–6, 7–11, 12–17, 18–24, 25–44, 45–64, and 65–74. For female population, the menstruating and menopausal populations were identified as follows: Between 15 and 24 years old, all women are menstruating, between 25 and 64 there are both menstruating and menopausal women and from 65 onwards there are only menopausal women. In addition, for adolescent females, two age classes were identified: 12–14 which were considered non menstruating, and 15–17 which were considered as menstruating.

**Figure 1:**
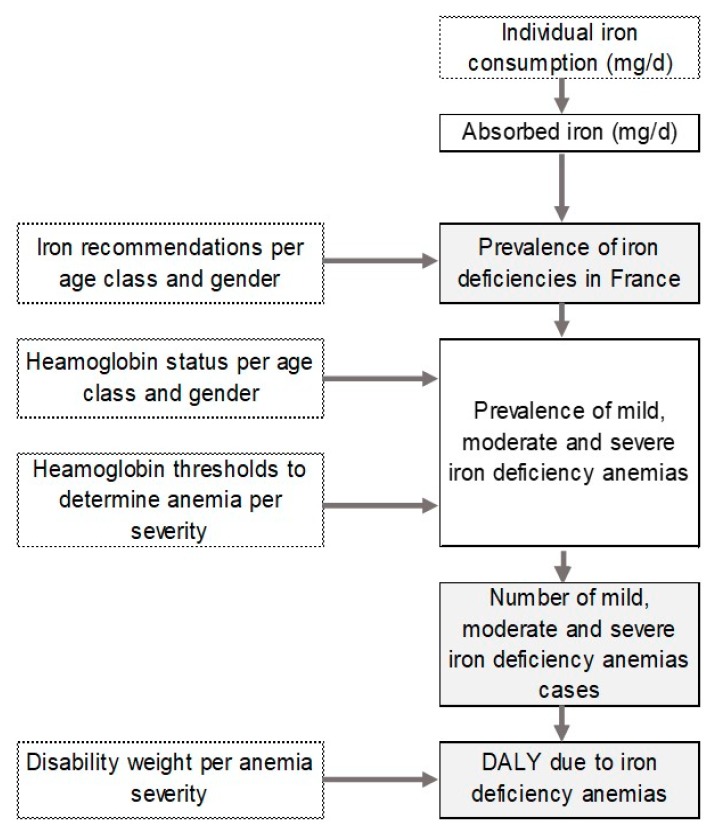
Flowchart of the assessment model of iron deficiency anemia disease per year in France per age class and gender. White rectangles with dashed line correspond to the “Inputs”, full line to “Intermediate calculation”. Light grey rectangles correspond to the “Final output”. Absorbed iron corresponds to the mean absorbed values provided by European Food Safety Authority (EFSA) considering both heme and non-heme iron.

The flowchart of the risk assessment model is presented in Figure 1. From iron consumption in France, the mean absorption of iron, the iron recommendations, and hemoglobin level in the French population, the number of cases and then the burden of disease per year, gender, and age class were estimated.

When building the model, several assumptions were made and validated by a medical expert. Three hypotheses were considered:

- ID appears when iron requirements are not covered by dietary iron.
- The ratio between the proportion of ID and IDA remains constant over the consumption scenarios.
- The consumption of bioavailable dietary iron, such as that found in red meat, reduces IDA.

### 2.2. Iron Intake Consumption in France

Iron intake in metropolitan France was evaluated between 2005 and 2007 for males and females aged from 3 to 79, by the dietary survey INCA 2 (approved by the French National Commission for Computed Data and Individual Freedom, “Commission Nationale Informatique et Libertés”; CNIL, under the registration code 797859—v0, on 20 March 2002). From the original data available at https://www.data.gouv.fr/ [18], we selected all-male population and non-pregnant women whose information about pregnancy and menopausal status was available. For unknown status of adult women, this latter was deduced or hypothesized. When the answer about menopausal status was unknown, the pregnancy status was verified. If the latter was negative, the average age of menopausal occurrence was taken into account. Therefore, women aged over 50 years old were assumed menopausal. Women aged less or equal to 50 years old, were assumed pre-menopausal [19]. On the other hand, when pregnancy status was unknown, the menopausal status was verified. If the answer was negative, women were considered as pregnant. Pregnant women were excluded from the study because the number of cases was low for each age class concerned (under 30). Under 18 years old, women were considered not pregnant. From the iron consumed, only a certain amount was absorbed. As for European Food Safety Authority (EFSA) estimations, the absorbed iron from iron intake was 10% for children under 11 years old, 16% for adolescents and adult males, and 18% for adult females [20].

To estimate the probability density of iron intake, the function fitdist of the package fitdistrplus was used with the R software version 3.4.0. Based upon the Akaike information criterion (AIC), the lognormal distribution provided the best fit, among Weibull, normal, lognormal and gamma distributions.

### 2.3. Prevalence of Iron Deficiency in France

To calculate ID prevalence in France, iron intake from dietary survey INCA 2 (see Table 1 at “Iron consumption (absorbed)”), was compared with a normal distribution of iron needs, as suggested by the Nordic Council of Ministers in 2014 [21] based on values given by the European Food Safety Authority (EFSA) (2015) [20] (see Table 1 at “Iron needs”). The mean parameter of the normal distribution was the absorbed average requirement value, the standard deviation was the value directly provided by EFSA report, except for males under 17 and females under 14 for which the standard deviation was deduced from the confidence interval upper limit bound of the EFSA report. In the present study, adolescent girls were divided into two groups: Girls from 12 to 14 were considered having not a menstruating status—mean menarcheal age of 13 years old in France [22]—and girls aged from 15 to 17 were assumed to have the iron needs as adult women since all the individuals in this age class were assumed to be menstruating.

The prevalence of people consuming less than the normal probability distribution of the needs was then estimated, using plnorm function of R software. This population was considered as iron-deficient. By multiplying the prevalence of ID in France by the number of individuals for each age class and gender (see Table 1 at “French population data”), the number of ID cases in France per year was estimated.

### 2.4. Prevalence of Iron Deficiency Anemia in France

Anemia results from levels of hemoglobin <12–15 g/dL and 51% (21%–85%) of anemia is due to ID [10,12,23]. To estimate IDA prevalence from iron deficiency, we based our calculations on the reported iron deficiencies, iron deficiencies anemias, and anemias from three studies [24,25,26]. From ID, IDA were calculated as follows:

(1) $$Prev.IDA_{a,g}=Prev.ID_{a,g}\times Prop.IDA_{a,g}$$

where a is the age class, g the gender, Prev.IDA the prevalence of IDA, Prev.ID the prevalence of ID and Prop.IDA the proportion of IDA due to ID in France. The data are given in Table 1.

To determine the proportion of anemias per severity (mild, moderate, severe), the level of hemoglobin of the French population was considered. Stoltzfus et al., 2004 provided hemoglobin levels in blood of children in EUR-A WHO region. The standard deviation of hemoglobin taken into account in this study was 1.0 g/dL for countries with anemia prevalence under 15%, as it is in France [9,23] (see Appendix A.1 Table A1). In 2011, a study evaluated the iron status in European adolescents and found similar hemoglobin levels [27]. The following age class and hemoglobin levels were estimated from linear functions. Adult male and female hemoglobin levels were provided by Santé Publique France data from ENNS (Étude Nationale Nutrition Santé) study [26]. The methodology to determine hemoglobin levels of the French population is presented in Appendix A.1 and the levels used in the present study are given in Table 1 and in Appendix A.1 Table A2. These levels of hemoglobin followed a normal distribution from which the proportion of anemia in France (with and without ID) was estimated.

According to “The burden of diseases of anemia”, three levels of anemia were considered: Severe, moderate, and mild, as used by Kassebaum on the behalf of GBD 2013 Anemia Collaborators evaluation (2016) based on which health state descriptions are presented in Table 2.

The prevalence of anemia per severity, $Prev.IDA_{a,g,s}$, was then calculated as follows:

(2) $$Prev.IDA_{a,g,s}=Prev.IDA_{a,g}*Alloc.Ane_{a,g,s}$$

The allocation of anemia severity, among the anemia population, $Alloc.Ane_{a,g,s}$, is deduced from the hemoglobin normal distribution per age class and gender (Hb) in the French population and hemoglobin thresholds (Hb.t) defined by WHO [4]. The methodology is provided in the Appendix A Method A2. We assumed that the proportion of anemia per severity (with and without ID) in the French population was the same as for anemia only due to ID.

### 2.5. Burden of Diseases from Iron Deficiency Anemia

The burden of diseases of the outcome attributable to IDA was expressed in the number of cases and disability adjusted life years (DALY). This metric estimates an equivalent number of years in good health lost due to the outcome [30].

DALY estimation takes into account mortality by considering the YLL for the age class and gender concerned. In addition, morbidity is also included and expressed in YLD. YLD is obtained by multiplying the decrease in the quality of life, expressed by a disability weight factor due to the sequelae or the stage of the outcome, by the duration of the specific disability. The disability weight factor is estimated between 0 (perfect health state) and 1 (death), available in the GBD estimations [4,29].

DALY estimation was calculated for the French population per gender and age class and expressed per 100,000 people per year, for both outcomes.

Number of IDA per age class, gender and severity ($NB.IDA_{a,g,s}$) was calculated, considering the number of individuals in the concerned population ($Pop_{a,g}$):

(3) $$NB.IDA_{a,g,s}=Pop_{a,g}\times \;Prev.IDA_{a,g,s}$$

Then, the number of DALY due to IDA per age class and gender ($DALY_{a,g}$) was estimated as follows:

(4) $$DALY_{a,g}=\underset{s}{\sum }NB.IDA_{a,g,s}\times w_{a,g,s}$$

where w was the disability weight factor.

### 2.6. Second-Order Monte Carlo Simulation

To estimate the ID prevalence, two distributions were used: Iron intake and iron needs. The iron intake distribution came from a fitting procedure with bootstrapping to separate uncertainty and variability. The iron needs’ normal distribution was built using EFSA report values, it included only variability. The ID prevalence was then estimated by Monte Carlo simulation in two dimensions (MC2D). One thousand iterations were run to capture the uncertainty and 10,000 iterations for the variability. Note that once the ID prevalence was estimated with its uncertainty, the outputs of the model conveyed only uncertainty. To verify the stability of the outputs, three simulations were carried out for each age class and gender, a variation less than 1% for the DALY output was obtained.

### 2.7. Consumption Scenarios

To decrease ID and IDA for the most concerned population—all populations with IDA—consumption scenarios were performed by setting the consumption of a food product rich in iron, at different quantities. Red meat was chosen as food model because it is the richest source of heme-iron in developed countries [11], which is the most absorbable type of iron by human beings, with 20% to 30% of iron intake absorbed [10]. The consumption scenario was made for consumption of cooked ground beef with 15% of fat, which is the type of beef meat most consumed by the French population (51 g per week) [18], most acceptable by youngest populations and easiest to eat by elderly populations. From initial iron intake by French individuals involved in INCA 2 study, and taken into account in this study, the iron from unprocessed red meat (muscle of beef, pork, veal, horse, lamb) consumption was estimated from CIQUAL tables available at https://ciqual.anses.fr/ [31] (see Appendix A.2 Table A3). The amount of iron from red meat consumption was subtracted from the total iron intake for the consumption scenario 0g/d of red meat. The consumption of 25 g/d, 50 g/d, 75 g/d, 100 g/d of cooked ground beef 15% fat was applied, with an amount of 2.6 mg of iron per 100 g of meat and an average absorption of 25% [10]. The consumption scenario took the amount of iron from other dietary sources (without red meat) and the addition of the amount of ground beef into account.

## 3. Results

### 3.1. Prevalence and Number of Iron Deficiencies in France

The distributions of ID with current consumption of iron in France are shown in Table 3. The mean number of ID cases is represented with its 95% confidence interval.

Among children, the most concerned with ID were children aged from 7 to 11, with 20% (95% CI = 16%–24%) and 31% (95% CI = 27%–36%) ID in this age class for males and females, respectively. Adolescent females were also more concerned with ID compared to adolescent males (Table 3).

The higher impact of ID among females was also noted in adults’ age classes. Menstruating adult females aged from 18 to 24 were the most concerned with ID with 37% [95% CI = 33%–41%] of iron-deficient in this population group, corresponding to 1600 [95% CI = 1400–1700] IDs cases per 100,000 individuals. After this age class, the prevalence of ID decreases with increasing age.

### 3.2. Number of Iron Deficiency Anemias

In France, only children aged from three to six, older adolescent females, adult females and males aged from 25 to 44 years and from 65 to 74 suffered from IDA [25,26]. The distributions of IDA with current consumption of iron in France are shown in Table 4. The mean number of IDA cases is represented with its 95% confidence interval.

A total of 1290 [95% CI = 1230–1350] cases of IDA per 100,000 individuals per year was estimated in the French population.

Children aged from three to six, had almost the same number of IDA cases for males and females with 32 (95% CI = 21–45) and 33 (95% CI = 23–45) cases per 100,000 individuals per year, respectively. Among adolescents, only females aged from 15 to 17 are concerned with IDA. The number of IDA cases for this population was ten times higher than for females aged from three to six.

Adult females were significantly more concerned with IDA than males with 584 (95% CI = 558–612) IDA cases versus 237 (95% CI = 194–282) IDA cases per 100,000 individuals, respectively. The major number of cases concerned menstruating women aged from 15 to 17 with 404 (95% CI = 377–431) IDA cases per 100,000 individuals per year. The cases of IDA for menopaused females only concerned the last age class.

### 3.3. DALY Attributable to Iron Deficiency Anemias

The number of DALY per 100,000 individuals per year associated with IDA in France is given in Table 4. The mean number of IDA DALY is represented with its 95% confidence interval.

The major contributors to the burden from IDA were menstruating females aged from 25 to 44 with 6.5 (95% CI = 4.2–9.0) DALY associated with IDA per 100,000 individuals per year. Young children aged from three to six, had a low burden with an equivalent number of DALYs (0.3 (95% CI = 0.1–0.5) DALY per 100,000 per year). Older adolescent females, aged from 15 to 17, contributed with 4.0 (95% CI = 2.1–5.9) DALY per 100,000 individuals per year.

The estimated number of DALY associated with IDA was 16 (95% CI = 11–20) per 100,000 French individuals per year, from which more than 80% of the DALY were attributable to menstruating females.

### 3.4. Consumption Scenarios

**Figure 2:**
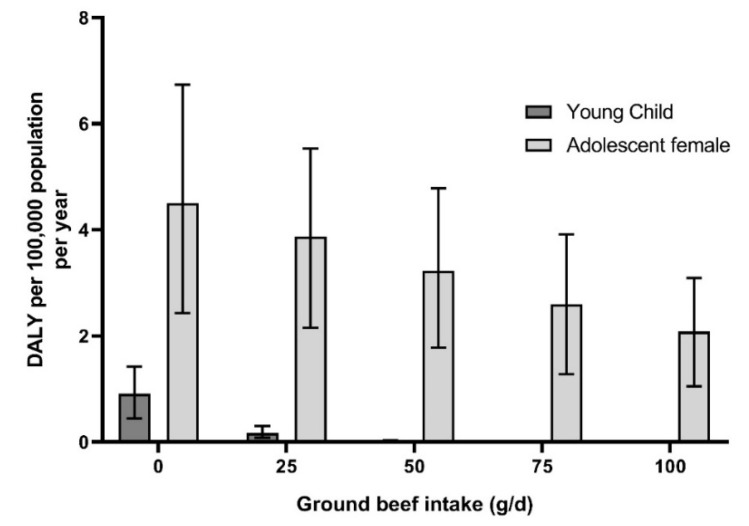
Estimated number of DALY from iron deficiency anemias in France for young children (three to six) and adolescent females (15–17) according to ground beef consumption scenarios. Results expressed per 100,000 individuals per year. Full lines represent the 95% uncertainty around the mean value.

**Figure 3:**
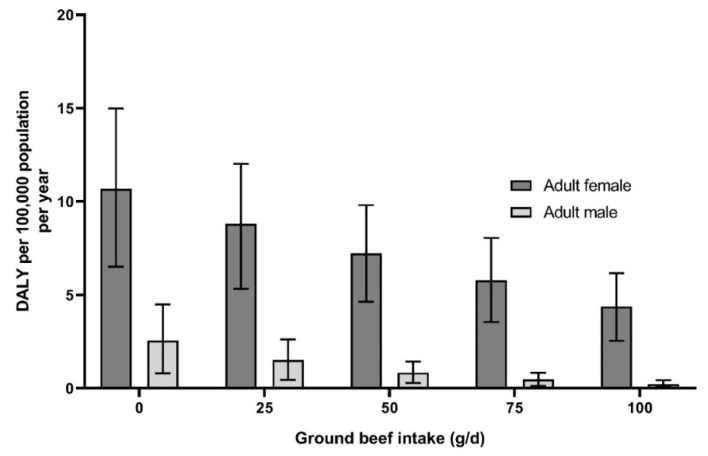
Estimated number of DALY from iron deficiency anemias in France for adult males and females according to ground beef consumption scenarios. Results expressed per 100,000 individuals per year. Full lines represent the 95% uncertainty around the mean value.

Based on the developed model, to estimate the effect of red meat intake on IDA burden of diseases, a consumption scenario for young children (three to six), older adolescent females (15–17), males (25–44; ≥60), adults menstruating females (≥18) and menstruated females (65–74) was generated. The results for young children and older adolescent females are presented in Figure 2 and results for adult females are reported in Figure 3.

For young children, the consumption of 50 g/d of ground beef, added to the iron consumption from other sources of iron without red meat, in current diet, would be enough to reduce the prevalence of ID to 0.2% (95% CI = 0%–1.9%) per 100,000 individuals per year and to decrease the burden of IDA close to zero DALY. For older adolescent females, the consumption of 100 g/d of red meat—which may represent a portion of red meat per day—added to the other sources of iron in current diet, would not be enough to eliminate ID and IDA. However, it would reduce the prevalence of ID to 22% (95% CI = 0%–90%) per 100,000 individuals per year and halve IDA burden when comparing with current consumption (mean red meat consumption of 18.2 g/d).

The burden of IDA in male adults was already low, but the consumption of 50 g/d or 100 g/d of red meat, in addition to the other sources of iron in current diet (without red meat), would decrease the current number of DALY from 0.8 [95% CI = 0.3–1.4] and 0.2 [95% CI = 0.1–0.4] per 100,000 individuals per year, respectively.

The burden of IDA in adult females aged over 18, were the highest in the population, especially for menstruating females (Figure 3). The consumption of 50 g/d or 100 g/d of red meat, in addition to the other sources of iron in current diet (without red meat) would decrease the current number of DALY down to 7.2 (95% CI = 4.6–9.8) and 4.4 (95% CI = 2.5–6.2) per 100,000 individuals per year, respectively.

## 4. Discussion

The present study objective was to build an original probabilistic assessment model to estimate the burden of diseases associated with ID and subsequently IDA with the current iron consumption. A probabilistic risk assessment model was developed to estimate the prevalence of iron deficiencies, the number of cases and DALY in France from iron consumption.

Our results were in line with a previous study in France, from 2001, especially for young children [25]. According to the study, 13.6% of young children aged from two to six were iron-deficient, which is not far from our estimations where 9% (95% CI = 6%–12%) of young boys and 9% (95% CI = 7%–13%) of young girls were iron-deficient. However, there is a missing population in this comparison. Due to the lack of consumption data, our estimations only included children aged from three to six. This estimation was also in line with iron deficiencies prevalence in Europe estimated between 7% and 18% for toddlers and young children [32]. On the other hand, these results were higher than those found in France in a more recent study which found 3.2% (95% CI = 2.0%–5.1%) [33], although the threshold for ID definition in this study was low with 10 or 12 µg/L—corresponding to the total depletion of iron storage. Indeed, the definition of ID was not the same because, with the data available, it was not possible to quantify the total depletion of iron, but the iron under the average requirement. For adolescents, it was estimated 15% (95% CI = 13%–18%) ID prevalence for males aged from 12 to 17 years old and 21% (95% CI = 17%–25%) and 44% (95% CI = 41%–47%) ID prevalence for adolescent females aged from 12 to 14 and from 15 to 17, respectively. This was in line with the estimations in Europe (between 0% and 43%) but was higher than iron deficiencies estimated for French adolescents (0% for males aged from 14 to 18, 3.1% for females aged from 11 to 15 and 15.4% for females aged from 14 to 18) [24]. Nonetheless comparison was hard to establish because age classes were not the same as those in this study, the data were from 1988 and the definition of ID threshold was not the same [34]. For the adult population, the most concerned with ID were menstruating adult females. This is mainly because iron losses are higher than for males and menopausal females, then, iron needs are higher for menstruating females (1.41 mg/day) compared to other adults’ needs (0.97 mg/day). In addition, iron consumption by adult menstruating females (mean 11 mg/d, sd 4 mg/d) was lower than by adult males (mean 14 mg/d, sd 5 mg/d). The values estimated in our study were similar to those measured in blood of French adults in ENNS study (for serum ferritin ≤30 µg/L) [35].

The estimated number of IDA cases was 1290 (95% CI = 1130–1350) IDA per 100,000 individuals per year (Table 4) with 584 (95% CI = 558–612) cases per 100,000 people for adult women per year. This corresponded to 797,200 (95% CI = 759,900–836,900) for the French population per year. The major contributors were menstruating females aged from 15 to 17. Indeed, twenty-two percent of older adolescent females were identified with IDA in our study, which was higher than the results found in 1994 in France (7.4% for adolescents aged from 14 to 18) [24,34]. The relevance of this comparison is somewhat questionable as there is a substantial time difference between the two studies: 2007 versus 1994. For adult females, it was estimated 1.7% (95% CI = 1.6%–1.8%) of IDA in France which was lower than results obtained in the SUpplementation en VItamines et Minéraux AntioXydants (SU.VI.MAX) study (4.4%) [36]. However, the latter study only included individuals aged from 35 to 60 years old.

To go further than the estimation of the prevalence and the number of cases, we have taken into account morbidity with the use of the composite metric DALY [37,38], which is the most used in risk and benefit assessment studies [30,39]. Here, only morbidity was considered, because there is no existing data about IDA fatalities in France. Three levels of IDA severity were taken into account: Mild, moderate, and severe. The sequelae (Table 2) of the different levels of IDA were assumed to be the same whatever the sub-population, and were based on the health status definitions from the GBD study [29]. This assumption may induce an underestimation of the IDA burden for children and adolescents. Indeed, GBD health status does not take into account possible disabilities such as neurodevelopment delay due to ID and IDA which may induce impaired learning abilities, memory skills, and behavioral issues [40,41]. The majority of the studies evaluating the beneficial effect of iron supplementation were carried out in children under three years old. However, this age is considered under our first age class. After this age, some studies found a beneficial effect of iron treatment in older anemic children on intelligence quotient, but not in non-anemic [42]. Moreover, there was no improvement in other disabilities, such as academic achievement, memory, or motor development [42]. Therefore, experts remain cautious about these conclusions and more studies are needed to conclude about this effect. The estimated DALY of IDA cases was 16 (95% CI = 11–20) per 100,000 people per year (Table 4) which corresponds to 9580 (95% CI = 7050–12,250) DALY for the French population per year. These values were lower than, but of the same order of magnitude as the estimation by GBD’s study which reported 35 DALY per 100,000 French population in 2007 [43]. The major contributors to the burden were females aged from 25 to 44 and no longer adolescent girls aged from 15 to 17. This was due to the higher prevalence of moderate and severe anemia cases in the adult age class. However, our estimations were underestimated because children under three years old and pregnant females, which are particularly vulnerable populations, were excluded from the study [4,5].

The quantification of variability and uncertainties, as recommended by international organizations [44], took into account the fitted distribution of iron intake and disability weight. For the first input cited, uncertainty and variability were quantified separately. However, for the second input cited, this separation was not possible. Even if some classify it as only variability [45]—the confidence interval representing the variability of the severity of the disability—we decided to classify it as only uncertainty as done by the majority of the previous studies [46,47]. The variability of the iron needs and hemoglobin status were also taken into account in the study.

Nonetheless, despite the inherent uncertainty and variability described above, the results obtained in this study can be interpreted as showing that some differences in the burden of disease were observed, such as between males and females, or between menopausal and menstruating females.

The use of DALY metric also enabled to make consumption scenarios for concerned populations. Due to the richest source of heme-iron—which is the form of iron most absorbable by humans—brought by red meat consumption [11], ground beef was taken as a food item in intake scenarios. In addition, consumption of red meat was found to increase the level of serum ferritin in the blood [12,13]. It was estimated that for young children, 50 g/d of ground beef—corresponding to 1.3 mg of iron—added to the other sources of iron already present in current diet (without red meat), would be enough to almost eliminate the IDA burden. For females aged over 15 years old and adult males, the consumption of 100 g/d of ground beef—corresponding to 2.6 mg of iron—added to the other sources of iron already present in current diet (without red meat) would not be enough to eliminate IDA burden (Figure 2; Figure 3). These results were in line with French Pediatric Society recommendations of eating 100–150 g/d of meat for children under six years old who stopped increasing milk consumption and twice a day by children over seven years and adolescents [10], since beef meat, which has the highest level of heme-iron, is not the only meat consumed. The amount required to suppress IDA for adults is above the French Agency for Food, Environmental and Occupational Health and Safety (ANSES) recommendations, especially for adult women, which is to consume less than 500 g per week of red meat [48]. This recommendation was largely based on the conclusions of the World Cancer Research Found/Imperial College of London and the WHO, which classified red meat as “probably carcinogenic to humans” for colorectal cancer [49,50]. In addition, a recent study of our team determined that the consumption of over 65 g/d of red meat would increase the risk of cardiovascular disease for the most concerned populations (adult population, especially elderly) [51]. Indeed, heme-iron, is suspected to be the main component in the mechanism associated with colorectal cancer [52,53] and cardiovascular disease [54,55,56]. These latter health effects were not considered to be of concern for children and adolescents for whom current epidemiological data were available [57], but more studies and meta-analysis are needed to confirm this lack of association.

Furthermore, the intake scenario in this study does not consider the substitution and the acceptability of red meat. Indeed, when the individual increases the consumption of one food item, he/she may reduce the consumption of other foods. In addition, some population group will not accept to consume more red meat due to organoleptic reasons, beliefs, or ethics (e.g., vegetarian diet). In these cases, other sources of iron can be considered to reduce ID and IDA. The consumption of iron-rich sources such as white beans (7.97 mg of iron per 100 g), breakfast cereals (7.03 mg of iron per 100 g), or lentils (6.51 mg of iron per 100 g) [31] would be a possible solution, especially for adult populations with a risk of colorectal and cardiovascular disease. Nevertheless, the quantity consumed of these other sources of iron, which are in form of non heme-iron, should be higher than that needed for red meat, because the degree of absorption is lower than for heme-iron products. Otherwise, some other heme-iron containing products, such as fish and poultry have a lower proportion of iron in the food compared to red meat. Some studies show that the vegetarian and vegan population presented a higher risk of iron deficiencies [58,59,60]. However, there are large variations of non heme-iron absorption (0.7%–22.9%). Indeed, when blood levels had low serum and plasma ferritin concentrations, the absorption of iron increased [61]. In addition, dietary enhancers (e.g., ascorbic acid, alcohol) and inhibitors (e.g., calcium, tannins, and polyphenols) influence the absorption of non heme-iron [62].

Another possible solution for ID decrease is iron supplementation. It was observed that early iron supplementation of low birth weight infants would reduce the prevalence of behavioral problems and linear growth [3]. Another study showed that iron supplementation with vitamin C of IDA children aged from 6 to 12 in Turkey, increased the intelligence quotient by 4.8 points [63]. It was shown to improve learning and memory in non-anemic ID adolescent girls, in adults with IDA, and that iron supplementation can partially reverse the effects on cognitive performance.

## 5. Conclusions

The burden of the consumption of iron has been estimated and expressed in terms of prevalence, number of cases, and DALY. The study estimated 13,630 (95% CI = 5740–23,510) ID cases per 100,000 individuals per year. In terms of IDA, this corresponded to 1,290 (95% CI = 1230–1350) IDA cases per 100,000 people per year and to 16 (95% CI = 11–20) DALY per 100,000 individuals per year. In a further study, the IDA burden estimated here in terms of DALY will be balanced with other risks of iron-rich food intake, such as red meat. Indeed, for children and adolescents, the benefit of decreasing iron deficiency anemia can be compared with risks due to microbiological foodborne illnesses when eating red meat [64]. In addition to this latter risk, for adult populations, colorectal cancer and cardiovascular diseases attributable to red meat consumption [51,65] will be also considered in this overall risk–benefit assessment.

## Figures and Tables

**Table 1:** Sources of information and implementation of the inputs either as deterministic values or as probability distributions.

Characteristic		Input	Gender ^1^	Model Implementation Per Age Class ^2^								Unit	Type ^3^	From
				3–6	7–11	12–14	15–17	18–24	25–44	45–64	65–74			
French population data		Pop	Male	1,571,427	1,925,359	2,352,805		2,788,141	8,279,094	7,663,979	2,269,631	number	D	[28]
			Female Pre-M	1,498,259	1,829,236	1,084,687	1,159,862	2,649,398	7,910,584	2,694,011	-			
			Female Post-M	-	-	-	-	-	157,425	5,313,189	2,712,349			
Iron consumption (absorbed)			Male	$LogN\left(\begin{array}{c} -0.21\;\\ \left[\begin{array}{c} 95\%\;CI=-0.26\\ -\left(-0.17\right) \end{array}\right],\\ 0.31\;\\ {}[95\%\;CI=0.27\\ -0.36] \end{array}\right)$	$LogN\left(\begin{array}{c} 0.06\;\\ \left[\begin{array}{c} 95\%\;CI=0.01\\ -0.09 \end{array}\right],\\ 0.28\;\\ {}[95\%\;CI=0.26\\ -0.31] \end{array}\right)$	$LogN\left(\begin{array}{c} 0.62\\ \;\left[\begin{array}{c} 95\%\;CI=0.59\\ -0.66 \end{array}\right],\\ 0.34\\ \;[95\%\;CI=0.31\\ -0.36] \end{array}\right)$		$LogN\left(\begin{array}{c} 0.52\\ \;\left[\begin{array}{c} 95\%\;CI=0.46\\ -0.58 \end{array}\right],\\ 0.37\\ \;[95\%\;CI=0.33\\ -0.42] \end{array}\right)$	$LogN\left(\begin{array}{c} 0.75\\ \;\left[\begin{array}{c} 95\%\;CI=0.71\\ -0.78 \end{array}\right],\\ 0.31\\ \;[95\%\;CI=0.29\\ -0.34] \end{array}\right)$	$LogN\left(\begin{array}{c} 0.74\\ \;\left[\begin{array}{c} 95\%\;CI=0.71\\ -0.78 \end{array}\right],\\ 0.32\\ \;[95\%\;CI=0.30\\ -0.34] \end{array}\right)$	$LogN\left(\begin{array}{c} 0.79\\ \;\left[\begin{array}{c} 95\%\;CI=0.75\\ -0.84 \end{array}\right],\\ 0.34\\ \;[95\%\;CI=0.31\\ -0.37] \end{array}\right)$	mg/day	U and V	[18]
			Female Pre-M	$LogN\left(\begin{array}{c} -0.29\;\\ \left[\begin{array}{c} 95\%\;CI=-0.34\\ -\left(-0.25\right) \end{array}\right],\\ 0.25\;\\ {}[95\%\;CI=0.21\\ -0.28] \end{array}\right)$	$LogN\left(\begin{array}{c} -0.07\;\\ \left[\begin{array}{c} 95\%\;CI=-0.10\\ -\left(-0.03\right) \end{array}\right],\\ 0.29\;\\ {}[95\%\;CI=0.26\\ -0.31] \end{array}\right)$	$LogN\left(\begin{array}{c} 0.43\;\\ \left[\begin{array}{c} 95\%\;CI=0.38\\ -0.48 \end{array}\right],\\ 0.34\;\\ {}[95\%\;CI=0.30\\ -0.37] \end{array}\right)$	$LogN\left(\begin{array}{c} 0.40\;\\ \left[\begin{array}{c} 95\%\;CI=0.36\\ -0.45 \end{array}\right],\\ 0.36\;\\ {}[95\%\;CI=0.33\\ -0.40] \end{array}\right)$	$LogN\left(\begin{array}{c} 0.52\\ \;\left[\begin{array}{c} 95\%\;CI=0.46\\ -0.58 \end{array}\right],\\ 0.37\\ \;[95\%\;CI=0.33\\ -0.42] \end{array}\right)$	$LogN\left(\begin{array}{c} 0.59\;\\ \left[\begin{array}{c} 95\%\;CI=0.56\\ -0.62 \end{array}\right],\\ 0.35\;\\ {}[95\%\;CI=0.33\\ -0.37] \end{array}\right)$	$LogN\left(\begin{array}{c} 0.67\;\\ \left[\begin{array}{c} 95\%\;CI=0.62\\ -0.73 \end{array}\right],\\ 0.39\;\\ {}[95\%\;CI=0.35\\ -0.43] \end{array}\right)$	-			
			Female Post-M	-	-	-	-	-	$LogN\left(\begin{array}{c} 0.19\;\\ \left[\begin{array}{c} 95\%\;CI=-0.09\\ -0.47 \end{array}\right],\\ 0.43\;\\ {}[95\%\;CI=0.25\\ -0.63] \end{array}\right)$	$LogN\left(\begin{array}{c} 0.64\;\\ \left[\begin{array}{c} 95\%\;CI=0.60\\ -0.68 \end{array}\right],\\ 0.38\;\\ {}[95\%\;CI=0.35\\ -0.41] \end{array}\right)$	$LogN\left(\begin{array}{c} 0.58\;\\ \left[\begin{array}{c} 95\%\;CI=0.53\\ -0.64 \end{array}\right],\\ 0.34\;\\ {}[95\%\;CI=0.30\\ -0.38] \end{array}\right)$			
Iron needs		IR	Male	N(0.5, 0.1)	N(0.8, 0.16)	N(1.27, 0.25)		N(0.97, 0.38)	N(0.97, 0.38)	N(0.97, 0.38)	N(0.97, 0.38)	mg/day	V	[20]
			Female Pre-M	N(0.5, 0.1)	N(0.8, 0.16)	N(1.13, 0.23)	N(1.41, 0.76)	N(1.41, 0.76)	N(1.41, 0.76)	N(1.41, 0.76)				
			Female Post-M	-	-	-	-		N(0.97, 0.38)	N(0.97, 0.38)	N(0.97, 0.38)			
Proportion of iron deficiencies anemia when iron deficiency		Prop.IDA	Male	0.147	0	0		0	0.26	0	0.42	number	D	[25,26]
			Female Pre-M	0.147	0	0	0.5	0.05	0.09	0.10				
			Female Post-M	-	-	-	-	-	0	0	0.03			
Hemoglobin status		Hb	Male	N(12.8, 1)	N(13.3, 1)	N(13.9, 1)		N(15.5, 0.8)	N(15.3, 1)	N(15.3, 1.1)	N(14.9, 1.9)	g/dL	V	[23,26]
			Female Pre-M	N(12.7, 1)	N(13.4, 1)	N(13.4, 1)	N(13.4, 1)	N(13.5, 1.5)	N(13.5, 1.4)	N(13.7, 1.1)				
			Female Post-M	-	-	-	-	-	N(13.8, 0.6)	N(13.8, 0.9)	N(14, 1.2)			
Hemoglobin threshold for anemia severity	mild	Hb.t	Male	10.9	11.4	11.9	11.9	12.9	12.9	12.10	12.9	g/dL	D	[4]
			Female	10.9	11.4	11.9								
	moderate		Male	9.9	10.9	10.9				10.10	10.9			
			Female	9.9	10.9									
	severe		Male	7	8					9	8			
			Female	7	8									
Disability weight	mild	w	Both	N(0.005, 0.002)								number	U	[29]
	moderate		Both	N(0.058, 0.012)										
	severe		Both	N(0.164, 0.030)										

^1^ Female Pre-M, Premenopausal females; Female Post-M, Postmenopausal females. Menstruating females considered between 15 years and 64 years at most; ^2^ Following R parametrization; ^3^ D, deterministic; V, Variability; U, uncertainty.

**Table 2:** Iron deficiency anemia severity levels and health state descriptions from the Global burden of disease 2013 study [29].

Health State	Health State Description
Mild IDA	Feels slightly tired and weak at times, but this does not interfere with normal daily activities.
Moderate IDA	Feels moderate fatigue, weakness, and shortness of breath after exercise, making daily activities more difficult.
Severe IDA	Feels very weak, tired and short of breath, and has problems with activities that require physical effort or deep concentration.

**Table 3:** Prevalence and number of iron deficiencies per 100,000 French individuals, per age class and gender. Mean value and its 95% confidence interval.

Age Class	Male		Female			
			Premenopausal		Postmenopausal	
	Prevalence	Number of Cases	Prevalence	Number of Cases	Prevalence	Number of Cases
3–6	9% (6%–12%)	220 (140–310)	9% (7%–13%)	230 (160–300)	-	-
7–11	20% (16%–24%)	620 (510–730)	31% (27%–36%)	930 (810–1100)	-	-
12–14	15% (13%–18%)	580 (490–680)	21% (17%–25%)	360 (290–440)	-	-
15–17			43.7% (41%–47%)	820 (760–880)	-	-
18–24	9% (6%–12%)	400 (280–530)	37% (33%–41%)	1600 (1400–1700)	-	-
25–44	5% (4%–7%)	720 (580–880)	32% (30%–34%)	4100 (3900–4400)	34% (17%–50%)	90 (40–130)
45–64	4% (3%–4%)	450 (360–460)	27% (24%–31%)	1200 (1100–1300)	8% (7%–10%)	720 (580–860)
65–74	3.4% (2%–5%)	130 (80–190)	-	-	10% (7%–12%)	420 (320–530)

**Table 4:** Number of cases and disability adjusted life years (DALY) per 100,000 individuals of iron deficiencies anemias in France, per age class and gender. Mean value and its 95% confidence interval.

Age Class	Male		Female			
			Premenopausal		Postmenopausal	
	Number of Cases	DALY	Number of Cases	DALY	Number of Cases	DALY
3–6	32 (21–45)	0.3 (0.1–0.5)	33 (23–45)	0.3 (0.1–0.5)	-	-
7–11	0 (0–0)	0 (0–0)	0 (0–0)	0 (0–0)	-	-
12–14	0 (0–0)	0 (0–0)	0 (0–0)	0 (0–0)	-	-
15–17			404 (377–431)	4.0 (2.1–5.9)	-	-
18–24	0 (0–0)	0 (0–0)	77 (69–85)	1.6 (1.0–2.1)	-	-
25–44	183 (146–224)	0.9 (0.1–1.8)	374 (353–394)	6.5 (4.2–9.0)	0 (0-0)	0 (0–0)
45–64	0 (0–0)	0 (0–0)	122 (108–136)	1.2 (0.7–1.8)	0 (0–0)	0 (0–0)
65–74	54 (33–80)	0.6 (0.3–1.0)	-	-	11 (9–15)	0.1 (0.1–0.2)

## Appendix A

### Appendix A.1. Determination of the Hemoglobin Status in the Population

**Table A1:** Hemoglobin levels values of French population expressed in mean and (standard deviation) extracted from Stoltzfus et al. (2004) and Santé Publique France 2006–2007.

Age Class	Gender (and Status)	Hemoglobin (g/dL)	Reference
0–4	Male	12.5 (1)	[23]
	Female	12.5 (1)	
5–14	Male	13.4 (1)	
	Female	13.4 (1)	
15–29	Male	14.6 (1)	
	Female	13.4 (1)	
18–24	Male	15.5 (0.8)	[26]
	Female (Premenopausal)	13.5 (1.5)	
25–44	Male	15.3 (1)	
	Female (Premenopausal)	13.5 (1.4)	
	Female (Postmenopausal)	13.8 (0.6)	
45–64	Male	15.3 (1.1)	
	Female (Premenopausal)	13.7 (1.1)	
	Female (Postmenopausal)	13.8 (0.9)	
65–74	Male	14.9 (1.9)	
	Female (Postmenopausal)	14 (1.2)	

The levels of hemoglobin for the age classes under 18 years old (Table A1), did not correspond to the age classes used in this study. To determine hemoglobin status for the age classes 3–6, 7–11, 12–17 for male and 12–14 and 15–17 for females, the values were determined from linear functions, established from Stoltzfus et al. (2004) hemoglobin levels from Table A1.

Two linear functions were assumed to determine hemoglobin levels for females and one linear function for males aged under 18 years old:

- To determine female hemoglobin level in the age class three to six years old, the linear function was expressed:
  $$Hb_{a,g}=0.1173\times mean\;age\;of\;the\;class+12.275$$
- For females, age class from 7 to 11 and from 12 to 17 years old, the average hemoglobin levels were similar and estimated by the fallow function:
  $$Hb_{a,g}=-0.0015\times mean\;age\;of\;the\;class+13.413$$
- To determine male hemoglobin level in the age class 3–6, 7–11, and 12–17 years old, the linear function was expressed:
  (A1) $$Hb_{a,g}=0.1058\times mean\;age\;of\;the\;class+12.331$$

Hemoglobin distribution for each age class was assumed to follow a normal distribution. To determine the mean standard deviation for age classes under 18 years old, the standard deviation of hemoglobin considered in this study was the same as used by Stoltzfus et al. (2004) for countries with anemia prevalence under 15% (1.0 g/dL) [9,23]. For older age classes, the standard deviation was estimated from ENNS study mean and confidence interval.

The levels of hemoglobin used in the present study is given in Table A2.

**Table A2:** Hemoglobin levels of the French population expressed in mean and (standard deviation) used in the present study.

Age Class	Gender (and Status)	Hemoglobin (g/dL)
3–6	Male	12.8 (1)
	Female	12.7 (1)
7–11	Male	13.3 (1)
	Female	13.4 (1)
12–17	Male	13.9 (1)
12–14	Female	13.4 (1)
15–17	Female (Premenopausal)	13.4 (1)
18–24	Male	15.5 (0.8)
	Female (Premenopausal)	13.5 (1.5)
25–44	Male	15.3 (1)
	Female (Premenopausal)	13.5 (1.4)
	Female (Postmenopausal)	13.8 (0.6)
45–64	Male	15.3 (1.1)
	Female (Premenopausal)	13.7 (1.1)
	Female (Postmenopausal)	13.8 (0.9)
65–74	Male	14.9 (1.9)
	Female (Postmenopausal)	14 (1.2)

### Appendix A.2. Determining the Proportion of Anemia per Severity

To determine the proportion of anemia (Ane) per age class (a), gender (g), and severity (s), pnorm function was used from R software, considering hemoglobin distribution per age class and gender (Hb) in the French population (Table A2) and hemoglobin thresholds defined by WHO (Table 1) to define anemia per severity (Hb.t).

- Proportion of severe anemias:
  $$Ane_{a,g,s=severe}=pnorm\left[Hb.t_{s=severe},\;mean\left(Hb_{a,g}\right),\;standard\;deviation\left(Hb_{a,g}\right)\right]$$
- Proportion of moderate anemias:
  $$\begin{array}{l} Ane_{a,g,s=moderate}=pnorm\left[Hb.t_{s=moderate},\;mean\left(Hb_{a,g}\right),\;standard\;deviation\left(Hb_{a,g}\right)\right]-\\ Ane_{a,g,s=severe} \end{array}$$
- Proportion of mild anemias:
  $$\begin{array}{ll} Ane_{a,g,s=mild}= & pnorm\left[Hb.t_{s=mild},\;mean\left(Hb_{a,g}\right),\;standard\;deviation\left(Hb_{a,g}\right)\right]\\ & -\left(Ane_{a,g,s=severe}+Ane_{a,g,s=moderate}\right) \end{array}$$
- Allocation of anemia severity, among the anemia population
  $$Alloc.Ane_{a,g,s}=\frac{Ane_{a,g,s}}{\sum Ane_{a,g,s}}$$

**Table A3:** Quantities of iron in different types of red meat.

Red Meat Type	Iron (mg/100 g)
Cooked bourguignon beef	4.3
Cooked stew beef	4.3
Roasted horse meat	3.4
Roasted beef	3.1
Cooked ground beef 5% fat	2.83
Cooked ground beef 10% fat	2.71
Braised beef	5.9
Raw ground beef 5% fat	2.65
Cooked ground beef 15% fat	2.6
Grilled entrecote beef	2.55
Cooked ground beef 20% fat	2.48
Roast beef ribbon	2.4
Burgundy fondue	2.3
Beef grilled steak	2.21
Roasted lamb shoulder	2.2
Cooked dumplings beef	2.2
Beef carpaccio	1.89
Unspecified cooked meat	1.77
Mixed skewer of meat	1.77
Other meat	1.77
Lamb skewer	1.73
Beef skewer	1.41
Roast lamb	1.4
Cooked veal side	1.3
Roasted lean pork tenderloin	1.29
Grilled lamb chop	1.27
Braised ribs	1.2
Breaded veal cutlet	1.11
Roasted loin pork	1.09
Cooked escalope veal	1
Simmered veal	0.95
Roasted tenderloin veal	0.87
Roasted veal	0.87
Grilled pork chop	0.84
Roasted pork	0.64
